# A Randomised Controlled Trial Evaluating 3‐Year Survival Rates and Technical Complications of Screw‐Retained Hybrid Abutment Crowns on Two‐Piece Zirconia and Titanium Implants

**DOI:** 10.1111/clr.14443

**Published:** 2025-04-29

**Authors:** Guido Sterzenbach, Kristin Richter, Klara Alpen, Hediyeh Khoshreza, Florian Beuer, Theodor Thiele

**Affiliations:** ^1^ Department of Prosthodontics, Geriatric Dentistry and Craniomandibular Disorders Charité—Universitätsmedizin Berlin, Corporate Member of Freie Universität Berlin, Humboldt‐Universität zu Berlin and Berlin Institute of Health Berlin Germany; ^2^ MVZ Zahnmedizin Pankow, Dentalzentrum Pankow Berlin Germany

**Keywords:** ceramic implants, hybrid abutment crown, randomised controlled trial, screw‐retained, titanium, two‐piece implants, zirconia

## Abstract

**Objectives:**

This study compares the cumulative survival and technical complications of screw‐retained implant‐supported lithium disilicate crowns (SICs) on polyether ketone ketone (PEKK) base abutments and zirconia implants with those of titanium base abutments and titanium implants.

**Materials and Methods:**

Sixty participants were randomly and evenly assigned to receive zirconia or titanium implants. Survival and technical complications were assessed at 6 weeks after crown placement (baseline) up to 36 months. The cumulative survival of the SICs was analysed as a non‐inferiority design, assuming that the difference between the titanium group and the zirconia group is not more than 10%. Technical complications were assessed based on modified USPHS criteria and Pink Aesthetic Score (PES).

**Results:**

In the zirconia group, three early implant failures occurred; all of them were successfully revised. After baseline, three implants in the zirconia group were lost due to insufficient osseointegration, and therefore the SICs have to be categorised as failures even though none of the SICs failed. The non‐inferiority of the zirconia group could not be confirmed, as the cumulative survival was 10.7% lower compared to the titanium group (100%). The technical complication rate was low, with no statistically significant difference between the groups. The PESs improved significantly compared to baseline, with no significant difference between the groups at 12 months.

**Conclusion:**

Hybrid abutment SICs with PEKK base abutments on two‐piece zirconia implants could be an alternative to hybrid abutments SICs with titanium base on titanium implants. However, the lower osseointegration rate of the zirconia implants has to be considered.

**Trial Registration:** This study was registered in the German Clinical Trial Register (Deutsches Register Klinischer Studien) (number: DRKS00014866)

## 1 Introduction

Recently, two‐piece ceramic implant systems have been introduced in clinics as alternatives to corresponding titanium or one‐piece ceramic implant systems (Cionca et al. 2021; Koller et al. 2020). These two‐piece systems offer higher prosthetic flexibility to accommodate various clinical situations and facilitate guided bone regeneration, and withstand clinically relevant masticatory forces (Spies et al. 2016; Bethke et al. 2020). In contrast, one‐piece systems lack abutment angulation and have limitations in vertical positioning (Gamper et al. 2017; Kohal et al. 2023; Roehling et al. 2018).

Ceramic zirconium dioxide (ZrO_2_ or zirconia) is currently the most commonly used material for ceramic dental implants, abutments, and prosthetic reconstructions. Zirconia is a valid alternative to titanium due to its material properties and tooth‐like colour, particularly for single implant‐supported crowns (Soler et al. 2020; Biguetti et al. 2021).

With an increasing number of patients seeking metal‐free implant alternatives, zirconia is a valid alternative, providing fracture load behaviour to withstand masticatory forces and comparable soft‐tissue‐ and osseointegration behaviour to titanium (Comisso et al. 2021). In addition to its aesthetic advantages, zirconia shows less bacterial adhesion and plaque formation (Roehling et al. 2018; Sivaraman et al. 2018), potentially mitigating marginal bone loss due to peri‐implantitis. The reported survival rates of one‐piece and two‐piece zirconia systems are acceptable (Hashim et al. 2016; Padhye et al. 2023; Brunello et al. 2022; Sala et al. 2023).

In two‐piece systems with screw‐retained abutments, zirconia implants are combined with zirconia abutments or, in hybrid forms, with composite or polymer abutments such as polyether ether ketone (PEEK) or polyether ketone ketone (PEKK). These abutments are generally combined with adhesively bonded monolithic all‐ceramic crowns, such as those made of lithium disilicate (LS_2_). While sometimes cement‐retained, they are mostly screw‐retained (Joos et al. 2020), enabling damage‐free removal or restoration replacement as needed. Screw‐retained SICs minimise biological complications such as peri‐implant inflammation caused by cementation residues, which are responsible for issues seen with cemented abutments (Linkevicius and Vaitelis 2015; Cantarella et al. 2021; Kraus et al. 2022). Despite the benefits, screw retention can be challenging to realise and, depending on the design, handling, and clinical situation, may cause problems such as fractures or early loosening (Haro Adánez et al. 2018). In vitro studies on two‐piece screw‐retained ceramic implants have shown higher fracture rates at the abutment screw than two‐piece titanium implants (Preis et al. 2016; Osman and Swain 2015). To compensate for this, hybrid abutments made of thermoplastic polymers (PEEK or PEKK) have been designed to help mitigate stress shielding, as their elasticity modulus closely mirrors that of bone (Mishra and Chowdhary 2019; Perez‐Martin et al. 2021).

To date, no studies or randomised controlled trials have compared the cumulative survival and technical complications of all‐ceramic crowns on two‐piece screw‐retained zirconia‐based implant systems with their respective two‐piece titanium systems. In general, evidence directly comparing different materials in two‐piece systems is sparse (Neugebauer et al. 2023).

Recently adapted clinical guidelines, such as the EAO position paper or German S3 guidelines on the use of dental implants, have refrained from reaching a conclusion or recommending the use of two‐piece ceramic implants in clinical practice due to insufficient clinical evidence (Thiem et al. 2022).

This study closes this gap by providing robust clinical evidence for the aforementioned missing data.

It is hypothesised that:

Screw‐retained implant‐supported lithium disilicate (LS_2_) crowns on polyether ketone ketone (PEKK) base abutments and zirconia implants are non‐inferior to screw‐retained lithium disilicate (LS_2_) crowns on titanium base abutments and titanium implants. Both are expected to exhibit similar cumulative survival and rate of technical complications of the superstructure up to 3 years after baseline.

## 2 Materials and Methods

### 2.1 Study Design and Population

Total of 60 participants (34 women) with a mean age of 44 years (range: 21–67 years) were included in this prospective randomised controlled study between January 2018 and September 2019.

Written informed consent was obtained from all the participants.

The study was approved by the ethical committee of the Charité‐Universitätsmedizin Berlin (EA4/041/18) and performed in accordance with the Declaration of Helsinki, German Medical Device Law, ISO14155 Clinical Investigation of Medical Devices, and Good Clinical Practice. It was registered in the German Clinical Trial Register (DRKS00014866) and followed CONSORT guidelines.

#### 2.1.1 Inclusion/Exclusion Criteria

Eligible participants were required to be older than 20 years, have a minimum premolar or molar single‐tooth gap of 7.5 mm, and have a functional antagonist in the posterior region for the replacement unit. Implant placement requires acceptable bone volume and a residual bone height of no less than 10 mm to ensure implant placement without needing vertical or lateral bone augmentation. A waiting period between extraction and implantation of at least 4 months was required to achieve sufficient bone healing. Any implant adjacent to the planned implantation site had to be placed for at least 1 year.

Participants who were heavy smokers (> 10 cigarettes per day) or had a history of alcohol or drug abuse with the need for medical care were excluded from the study. Participants with systemic diseases, such as unmanaged or poorly managed diabetes, endocrine disorders, and cardiovascular disorders, and those taking medications known to affect bone metabolism, including bisphosphonates, corticosteroids, monoclonal antibodies, tyrosine kinase inhibitors, and methotrexate, were not considered. Other exclusion criteria included a history of head and neck irradiation, coagulopathy, or ongoing anticoagulation therapy, as well as substandard oral hygiene, active and untreated periodontal disease, parafunction such as bruxism with distinctive tooth wear, and known allergies or sensitivities to materials used in the study. Finally, this study did not include pregnant or lactating women.

Recruitment, treatment, and follow‐up of all participants took place at Dentalzentrum Pankow, Berlin, Germany.

#### 2.1.2 Randomisation

The participants were evenly randomised into two groups—the test group (zirconia group): zirconia implant, restored with a screw‐retained PEKK abutment with a bonded LS2 crown (Implant: CERALOG Hexalob 8 mm, 4.0, CAMLOG Biotechnologies GmbH, Basel, Switzerland; crown: IPS e.max CAD, Ivoclar, Lichtenstein)—and the control group (titanium group): titanium implant restored with a screw‐retained titanium adhesive base with a bonded LS2 crown (Implant: CAMLOG screw‐line 9 mm, 4.3, CAMLOG Biotechnologies, crown: IPS e.max CAD, Ivoclar). The allocation concealment was maintained by TT. The patients were allocated to one of the two study groups immediately before implantation by an individual not involved in the study through the random selection of one out of 60 lottery tickets from a sealed box (urn randomisation). The study was unblinded for the surgeons (TT and GS) due to the clear visual distinction between the two implant systems, while the clinicians who performed the clinical inspection at follow‐up assessment were blinded (KR, KA, HK).

### 2.2 Outcome Measures

#### 2.2.1 Primary Outcome

The cumulative survival and success of hybrid abutment SICs on two‐piece zirconia or titanium implants were assessed over a follow‐up period of up to 3 years. Survival was defined as partial or complete crown loss requiring a new SIC irrespective of the cause of failure.

#### 2.2.2 Secondary Outcome

The occurrence of technical complications of the SICs was evaluated using the modified USPHS criteria (Table 1) and aesthetic parameters, including the Pink Aesthetic Score (PES) and White Aesthetic Score (WES).

**TABLE 1:** Modified USPHS criteria for two‐piece screwed‐retained hybrid abutment ceramic SICs.

	A	B	C	D
Ceramic fracture	No chipping	Minor chipping	Major chipping	Large‐area fracture
Surface texture	Smooth	Slight roughness	Distinct roughness	Replacement
Anatomic form	Fits into dental arch	Slightly over‐/under‐contoured	Clearly over‐/under‐contoured	Clinically unacceptable
Aesthetic/colour	Good aesthetics	Slight colour difference	Strong colour difference	Clinically unacceptable
Occlusal closure colour	Good colour design	Colour difference	Discoloration	
Occlusal marginal gap	No gap	Gap detectable	Gap clearly visible	Fracture
Connection/retention	Firm	Loosening (screw, crown), clinically restorable	Loosening, restorable by replacing an element (screw, crown)	Replacement

### 2.3 Statistical Methods

The sample size was calculated based on an assumed cumulative survival rate of 95% for titanium SICs after 5 years.

The non‐inferiority limit (d) is set at 10%. The null hypotheses are therefore:

*π*_*t*_ *—π*_*k*_ *≥ 10% with πt and πz survival rates of titanium and zirconia groups, respectively, against*.

*πt—πz < 10%*.

0.05 (5%) is set for the probability of error of the first type alpha and 0.8 (80%) for the power to be achieved (1—beta). To test the zirconia group's non‐inferiority, 30 cases per group were required (Online Sealed Envelope Ltd. 2012 power calculator for binary outcome non‐inferiority trials; https://www.sealedenvelope.com/power/binary‐noninferior/ [Accessed Mar 02, 2018]).

In addition, the cumulative survival rates were determined using the Log‐Rank test.

Means and standard deviations were computed for descriptive analyses of the data. Appropriate tests (Fisher's exact test, exact Mann–Whitney *U* test, exact Wilcoxon test, and Log‐Rank test) were used to determine statistical significance (probability level, *p* < 0.05).

The primary endpoint analysis examined the cumulative survival rate of the final SIC, adhering to the intention‐to‐treat analysis (Kaplan–Meier calculation).

For the secondary endpoints for the USPHS criteria, PES and WES differences between groups at specified time intervals compared with baseline were analysed using the Mann–Whitney *U* and Wilcoxon tests.

All statistical tests and descriptive analyses were conducted using SPSS software (version 29).

### 2.4 Surgical Procedures Supported by Digital Workflow

**FIGURE 1:**
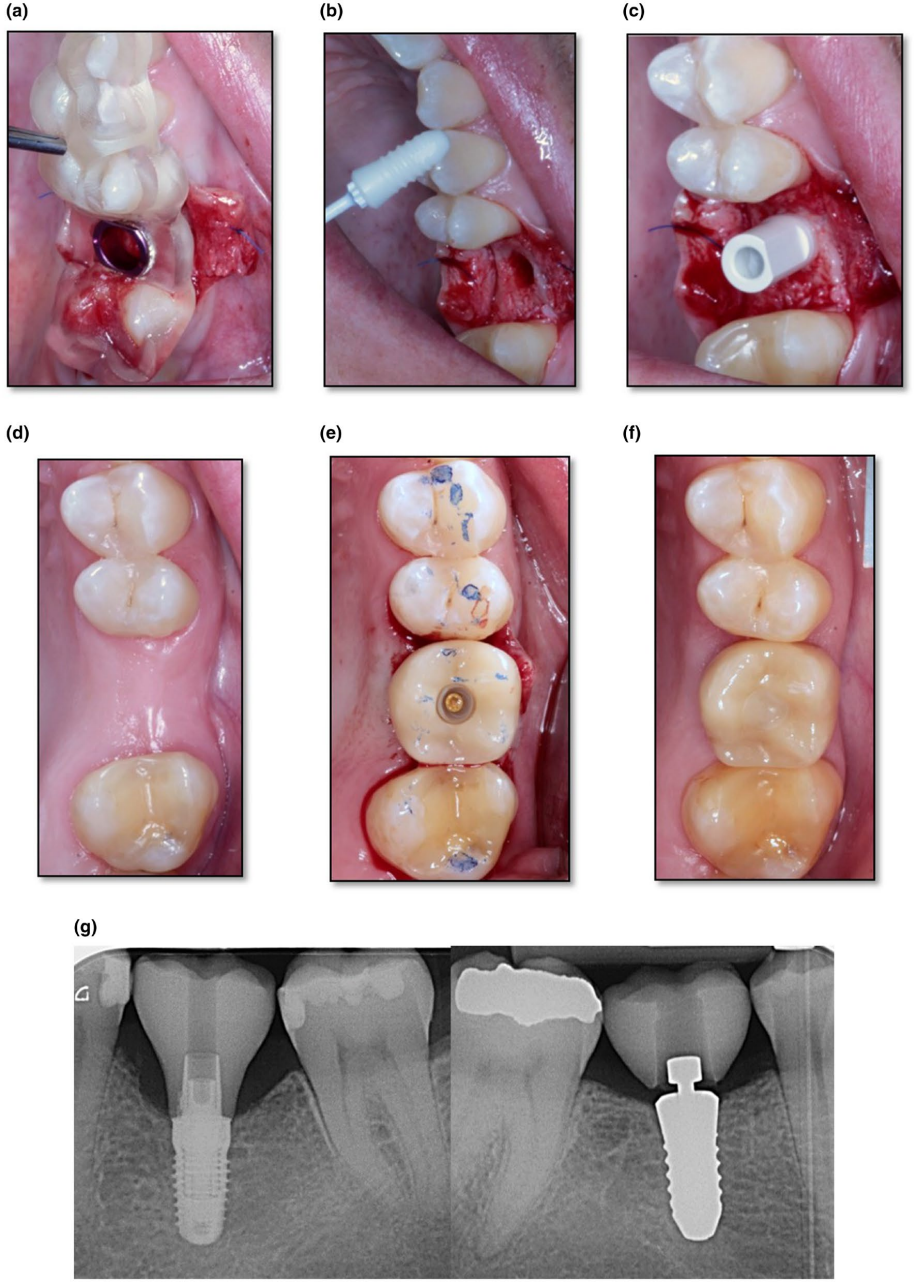
Clinical example of workflow (a) template for guided pilot drilling; (b) intraoral view before placement of CERALOG HL implant; (c) intraoperative Scanpost; (d) clinical situation after submerged Implant healing; (e) second‐stage surgery with split flap and final crown SIC of the CERALOG HL group; (f) finished SIC at baseline; and (g) radiograph of the finished SIC of the titanium and zirconia groups.

Implant positioning planning, drilling‐template fabrication, and hybrid abutment crown fabrication followed a digital workflow (see Figure 1).

#### 2.4.1 Implant Surgery

The semi‐navigated template‐guided implant placement was performed using backwards planning. A full‐arch scan using CEREC Omnicam (Cerec SW 4.6.1, Dentsply Sirona) was processed with the corresponding design software (InLab SW 18.1, Dentsply Sirona) to determine the ideal crown shape and position by superimposing imaging datasets (STL and DICOM data) in the corresponding planning software (Galileos Implant 1.9.2, SICAT, Dentsply Sirona). Drilling templates for correct implant positioning were manufactured using 3D printing (CAP‐Computer Aided Printing; V‐Print SG resin, SolFlex 170 SMP; VOCO GmbH, Cuxhaven, Germany).

The implants were inserted at the bone level under local anaesthesia (Ultracain 1:200000; Sanofi, Paris, France) according to the manufacturer's recommendations, starting with a crestal incision and mucoperiosteal flap creation to protect the soft tissue. The template helped to determine the maximum insertion length for pilot drilling, implant bed preparation (drill sequence via calibrated motor), and implant placement (motor and subsequent torque verification via hand ratchet).

Intraoperative intraoral scans of the implant position were performed using CEREC scan bodies with the CEREC Omnicam in group titanium. Since the manufacturer does not provide a scan body for the ceramic implants, the implant position of the zirconia implants was recorded using an intraorally fabricated splint made from light‐curing resin (Picobello, Picodent GmbH, Wipperfürth, Germany) prior to soft tissue closure. The splint was fixed to a printed model (V‐Print model, VOCO, SolFlex 170 SMP; VOCO GmbH, Cuxhaven, Germany) from the intraoral scan, and a model implant was screwed into place. The model scan of the PEKK base was then scanned with the CEREC Omnicam to fabricate the crown, as was done for titanium implants.

The titanium implants were fitted with cover screws, and the zirconia implants were sealed with sterile bone wax. The wound was closed with nonabsorbable suture material (Seralon 5/0, Serag‐Wiessner; Naila, Germany) and removed 7–10 days after surgery.

#### 2.4.2 Crown Manufacturing

Based on intraoral (group titanium) or model scans (group zirconia) LS_2_ SIC were fabricated from a lithium disilicate block, followed by individualisation through staining and glazing (IPS e.max CAD, Ivoclar) using computer‐aided design/computer‐aided manufacturing technology.

The bonding interface of the finished crowns was acid‐etched with hydrofluoric acid (VITA Ceramic etch 5%, VITA; Bad Säckingen, Germany) for 20 s, followed by priming with Monobond Plus (Ivoclar). The prefabricated abutments were pretreated with sandblasting (Al_3_O_2_ 50 μm, 4 bar) for both the PEKK‐base and titanium‐base or titanium‐luting bases. The crowns were bonded by hand using a luting composite (for the control group: Multilink, Ivoclar; and for the test group: Unicem 2, 3 M Deutschland GmbH, Germany).

#### 2.4.3 Restoration

The final restoration took place 4 months after ZrO_2_ and 3 months after Ti‐implant placement, respectively. Following local anaesthesia (Ultracain 1:200000; Sanofi), the implant shoulder was exposed, and subsequent soft tissue shaping to accommodate the individual crown shape was achieved via gap flap formation. Hybrid crowns were assessed for occlusal static contact with no dynamic contact during dynamic movements and aesthetics before the insertion of a titanium screw for the titanium implants and a gold screw for the zirconia group using a torque ratchet (25 Ncm for titanium and 15 Ncm for zirconia) as recommended by the manufacturer. After another torque check after 10 min, an occlusal seal of the screw channel was created with a composite (Amaris, VOCO GmbH, Germany) cured using a polymerisation lamp (3 M Elipar DeepCure‐S). Latero‐, mediotrusion, and static occlusion were re‐evaluated and adjusted using ceramic (Set‐1588 NTI CeraGlaze, NTI‐Kahla GmbH) and silicone polishers (Brownie and Greenie, Shofu Dental).

### 2.5 Follow‐Up Procedure

**FIGURE 2:**
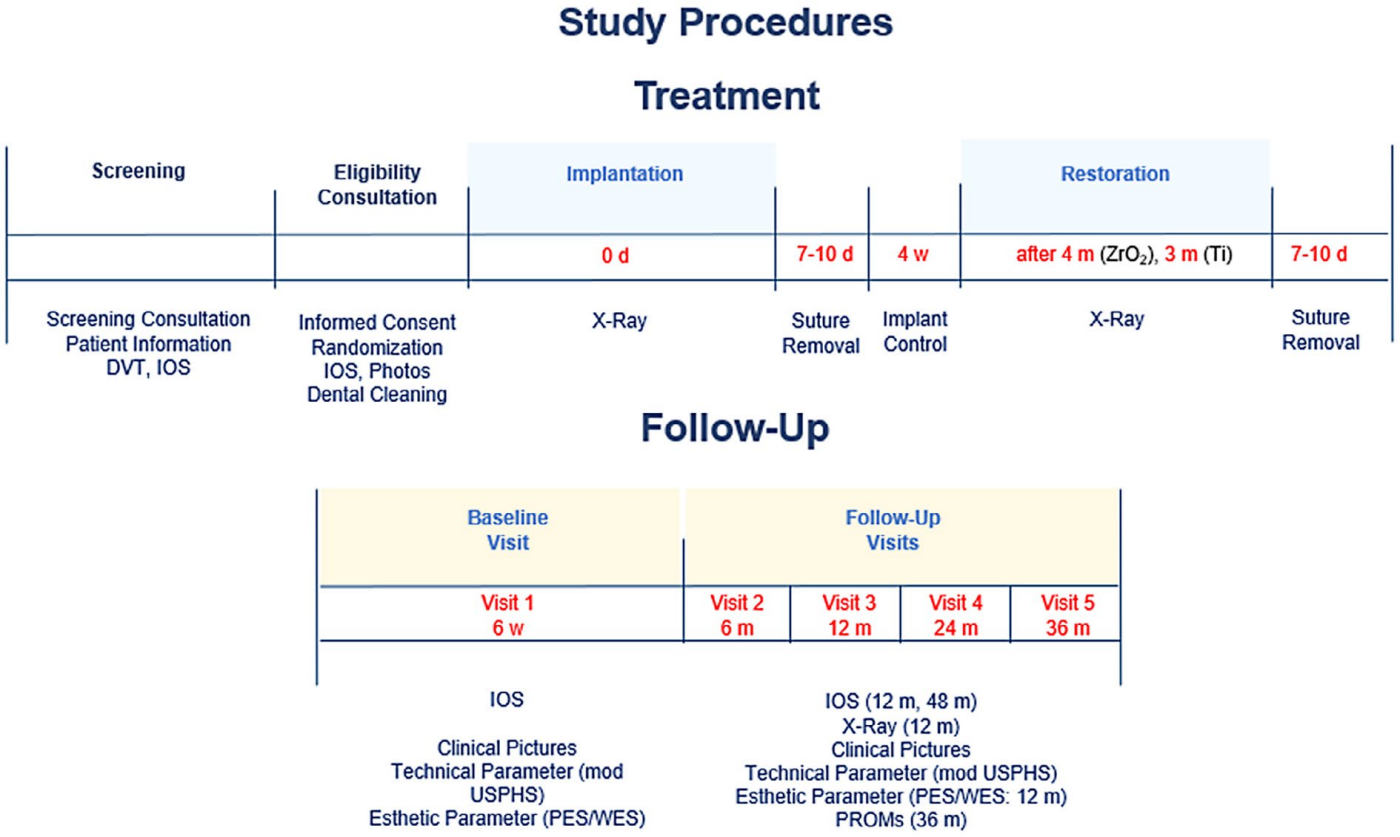
Study procedures (treatment and follow‐up). d, day; DVT, digital volume tomography; IOS, intraoral scan; m, months; n, number of participants; w, week. The input contains no grammatical issues and is unchanged.

SICs were evaluated according to the USPHS criteria for the first time at 6 weeks after crown placement to ensure complete soft tissue healing (baseline) and 6, 12, 24, and 36 months after baseline (Figure 2). PES and WES were evaluated at baseline and12 months.

### 2.6 Modified USPHS

Technical complications of the SICs were assessed using the modified USPHS criteria for all‐ceramic implant cemented crowns published by Spies et al. (2017) modified from United States Public Health Service (USPHS) criteria (Cvar and Ryge 2005). The modified USPHS used for cemented SICs was re‐modified to better suit the clinical situation of screw‐retained SICs. A scale from A to D, with A representing no problems to D stating significant issues, was used. Criteria included ceramic fracture, surface texture, anatomic form, aesthetics/colour, occlusal closure colour, occlusal marginal gap, and connection/retention of the crown on the respective customised abutment (see Table 1). The assessment was carried out at each recall appointment by trained practitioners (KR, HK, KA).

### 2.7 Pink Aesthetic Score/White Aesthetic Score

PES and WES were calculated as the sum of the scores of individual parameters, each ranging from 0 to 10, with 10 representing the highest quality, according to Fürhauser et al. (2005) and Belser et al. (2009). Scores of 6 or above were considered clinically acceptable.

The mean scores at baseline and at 12 months were assessed.

Three trained study staff members (KR, GS, HK) evaluated the clinical photographs for scoring. A value was deemed acceptable when at least two evaluators reached a consensus. Discussions were held in the event of differing assessments until a unanimous decision was reached.

### 2.8 Patient‐Reported Outcome Measures

In order to explore patient's perception on the treatment, an unvalidated questionnaire was developed by author's. This was intended to assess the entire treatment from the independent perspective of the study participants. The participants received the questionnaire without further explanations; answering was voluntary. The general question was: How satisfied are you with your implant restoration? The participants could choose from five possible answers ranging from very satisfied to very dissatisfied. Patient‐reported outcome measures (PROMs) covering the categories comfort, appearance, chewing function, taste perception, fit, and overall satisfaction were voluntarily administered after 36 months by sending the questionnaire to the participants by post.

## 3 Results

### 3.1 Study Population

No significant differences in age, sex, or time between implantation and SIC between the two study groups were observed (Table 2). Mandibular molars were the most frequently replaced teeth.

**TABLE 2:** Characteristics of the study population at baseline.

Baseline	Titanium	Zirconia	
Number	30	30	
Age (years) mean, range	43.8 (21–72.4)	44.1 (26.1–67.1)	+ ++
up to 35 years	8	7	‡
36–45	9	11	
46–55	9	7	
56+ years	4	5	
Gender (M/F)	12/18	12/18	
Baseline after restoration (days)	51.9	53	
Time after implantation (days)	194.9	196.4	
Implant region			
Lower jaw	20	24	‡
Upper jaw	10	6	
Left	15	12	‡
Right	15	18	
Premolar	8	6	‡
Molar	22	24	

Exclusions	Number of subjects	Reasons
Screening	9	Detailed in Figure 2
Lost to follow‐up (LTFU)	2	All efforts to locate failed
Implant failure	3	Failure through incomplete osseointegration

*Note:* +, Exact Mann–Whitney *U* test; ++, Exact two‐sample Komogorow–Smirnov test; ‡, Fisher´s exact test; ‘n.s.’ stands for not significant and means that all test results in the respective line were not significant.

### 3.2 Cumulative Survival of Ceramic SIC up to 3 Years

**FIGURE 3:**
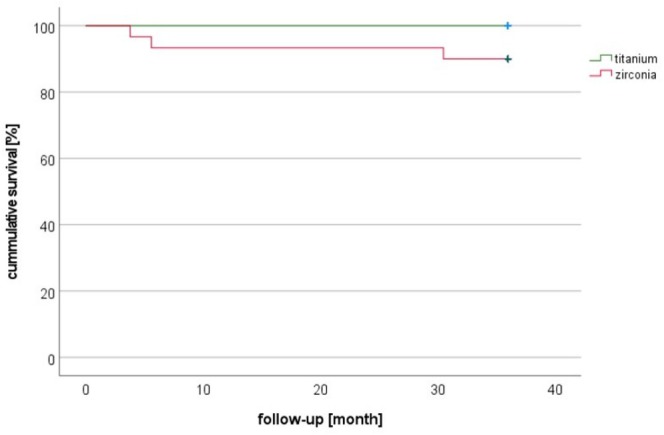
Kaplan–Meier cumulative survival.

**FIGURE 4:**
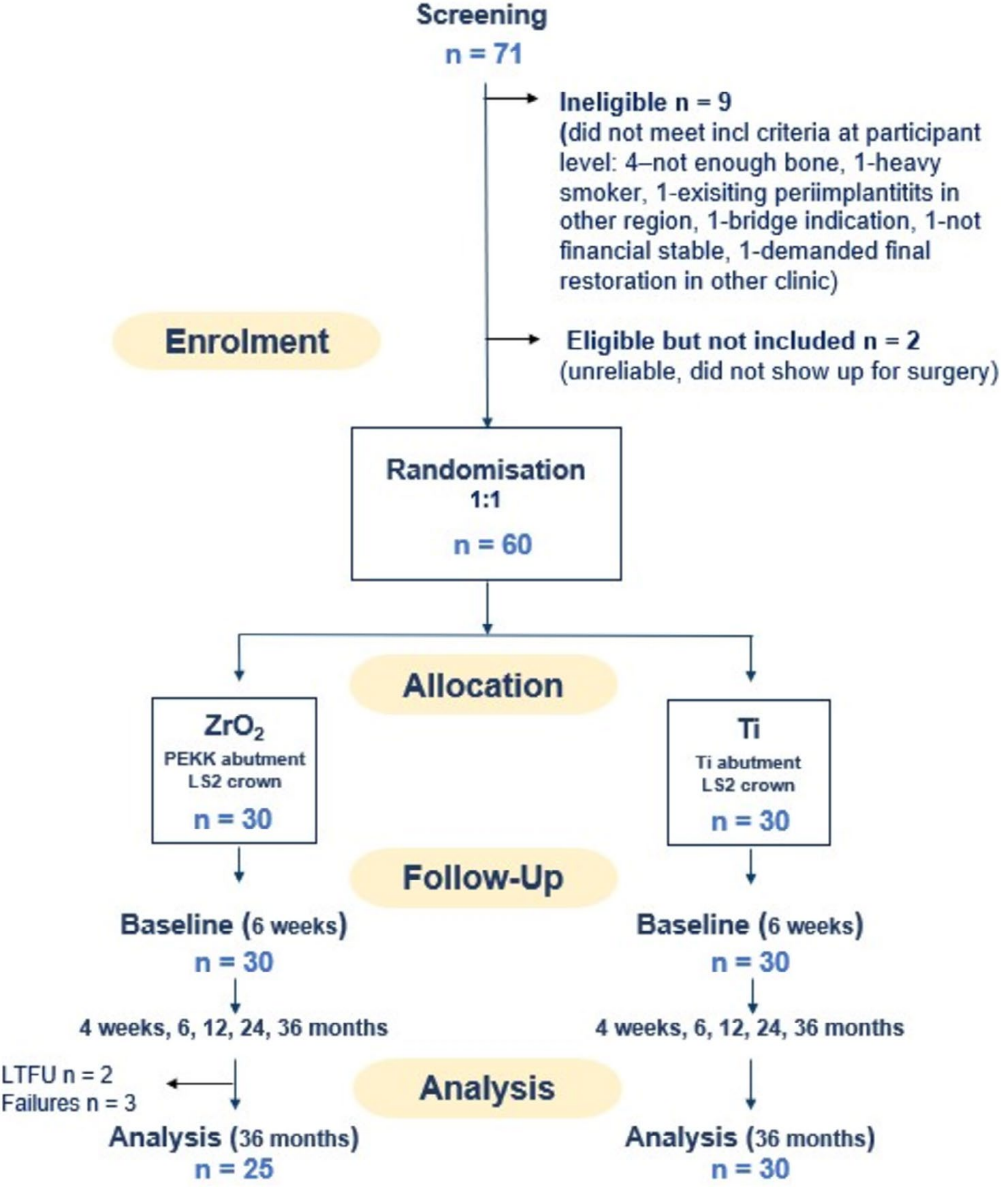
Study flow chart (CONSORT).

Three zirconia implants failed early, before any SIC could be performed. They were revised and healed without further complications, allowing the final treatment to proceed as scheduled. After baseline (final SIC), two failures occurred in the zirconia group during the first year and a third after 30 months (Figure 3). In all three cases, it was not the SIC that failed; the cause of the loss was a biological complication at the implant level. Aseptic, connective tissue encapsulation of the implants was observed in all three cases, indicating incomplete osseointegration. Furthermore, two participants in the zirconia group discontinued the study or failed to attend their follow‐up visits (Figure 4). All SICs in the titanium group survived. Thus, the cumulative survival rate of the zirconia group was 10.7% lower compared to the titanium group (100%).

Accordingly, the estimated cumulative survival rate (Clopper Pearson 90% confidence interval [CI], two‐sided (lower/upper)) after 3 years for SICs in the titanium group (*n* = 30) is 100% (0.926/1.0), while in the zirconia group (*n* = 28) it is 89.3% (0.746/0.97). Relative to the population, this represents a survival probability exceeding 74.6% with 95% confidence, one‐sided. The lower limit of the 90% CI for the two‐sided CI is the lower limit of the 95% CI for the one‐sided CI. In the worst case, the cumulative survival rate for the titanium group is 92.6% (lower limit of the one‐sided 95% CI). According to null hypotheses, the tolerance of 10% downwards means the cumulative survival rate of the test group (zirconia group) should be above 82.6%. The lower limit for the test group is 74.6% and is lower than this.

In contrast, the comparison of cumulative survival rates of SICs showed no significant difference between the groups (Log‐Rank test *p* = 0.068).

### 3.3 Evaluation of the Technical Parameters Using Modified USPHS Parameters

For all modified USPHS criteria, the differences in the assessment between the study groups after 36 months were tested using the exact Mann–Whitney *U* test. No significant differences were found in any of the evaluated parameters (Table 3).

**TABLE 3:** Modified USPHS parameters between study groups after 36 months.

Modified USPHS parameter	Implant material	A		B		C		Occurrence, *p* ^a^
		*N*	%	*N*	%	*N*	%	
Ceramic fracture	Titanium	29	96.7	1	3.3	0	0	0.202
	Zirconia	23	92.0	0	0	2	8	
Surface texture	Titanium	25	83.3	5	16.7	0	0	1.000
	Zirconia	20	80.0	5	20.0	0	0	
Anatomic form	Titanium	23	76.7	7	23.3	0	0	0.643
	Zirconia	19	76.0	5	20.0	1	4.0	
Aesthetic/colour	Titanium	19	63.3	11	36.7	0	0	0.501
	Zirconia	14	56.0	10	40.0	1	4.0	
Occlusal closure colour	Titanium	16	53.3	13	43.3	1	3.3	0.803
	Zirconia	13	52.0	12	48.0	0	0	
Occlusal marginal gap	Titanium	19	63.3	8	26.7	3	10.0	0.856
	Zirconia	16	64.0	8	32.0	1	4.0	
Connection/retention	Titanium	28	93.3	1	3.3	0	0	0.151
	Zirconia	23	92.0	2	8.0	2	8.0	

^a^ Exact non‐parametric Mann–Whitney U test.

No events were observed for category D (clinically unacceptable or requiring new fabrication of the SIC). Thus, we observed no technical failure in either group, but noted technical complications: In category C, which indicates the need for a repair of the existing SIC, we observed issues mainly related to chipping (two events in the zirconia group with no impact on clinical functionality) and loss of retention to the adhesive base (two events in the zirconia group which could be successfully re‐cemented). For category B, the following observations were made (group titanium/zirconia): anatomic form (23%/20%), aesthetic/colour (37%/40%), occlusal closure colour (43%/48%) and occlusal marginal gap (27%/32%). These mainly involved aesthetic deviations with no functional influence. Crown loosening due to screw loosening was recorded once in the titanium and twice in the zirconia group. In all three cases, the problem was solved by changing the screws. The rate of events in category B showed no significant difference between the groups. Therefore, the technical complication rate is not statistically different between the groups.

Accordingly, the categories “B” and “C” for the criteria “ceramic fracture” and “connection/retention” were assessed as requiring treatment, but without the need for a new restoration. In summary, two technical complications were observed in the titanium group and six in the zirconia group.

### 3.4 Evaluation of Aesthetic Parameters

#### 3.4.1 Pink Aesthetic Score

**FIGURE 5:**
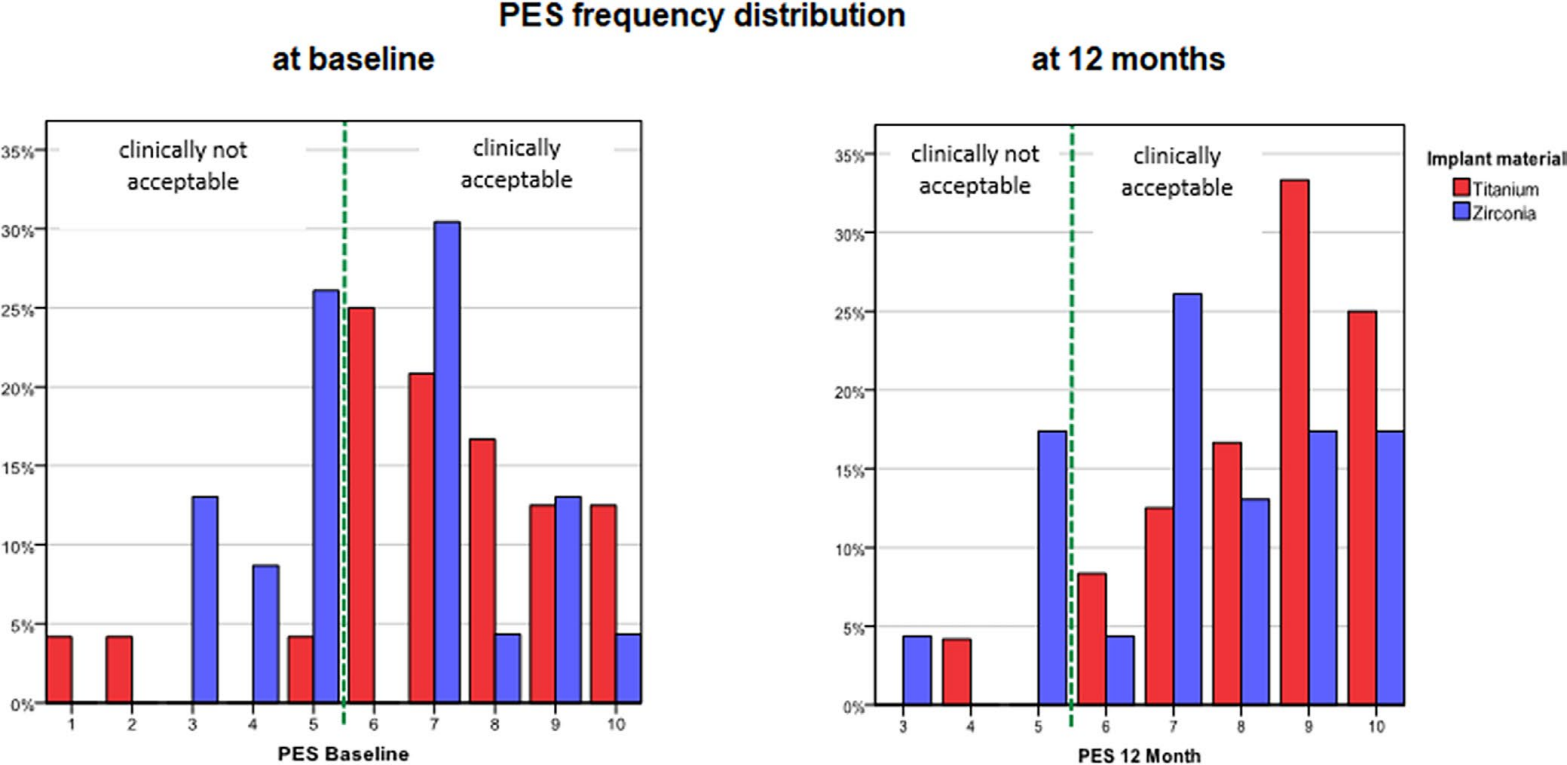
Pink Aesthetic Score (PES) frequency distribution at baseline and 12 months.

Notably, the zirconia group (mean: 6.13, SD 2.074) had a lower baseline level (Ti −12.5% vs. Zi, 47.8%, clinically unacceptable), which was significantly inferior to the titanium group (mean: 7, SD 2.226) in terms of the clinical acceptability of baseline PES values (*p* = 0.009, Fisher's exact test) (Figure 5). After 12 months, there was no significant difference between the two study groups in the average of PES (*p* = 0.085, Fisher's exact test). For both study groups, PESs improved significantly at 12 months (mean titanium: 8.38, SD: 1.555, *p* < 0.001; mean zirconia: 7.43, SD: 1.950, *p* < 0.001).

#### 3.4.2 White Aesthetic Score

The proportions of clinically acceptable values were identical at baseline.

There was no difference (mean; percentiles 25/75) between the zirconia group (7.65; 725/10) and the titanium group (8.33; 7/9) in the WES scores 12 months after baseline (*p* = 0.096, exact Mann–Whitney *U* test).

### 3.5 Patient‐Reported Outcome Measures

Since answering PROMs was voluntary, only 24 patients responded to the additional questionnaire (group titanium 50%, group zirconia 30%, Table 4).

**TABLE 4:** Patient‐reported outcome measures (PROMs) after 3 years.

	Implant material	1	2	3	4	5
		Very satisfied	Satisfied	Medium satisfied	Dissatisfied	Very dissatisfied
		*N* (%)	*N* (%)	*N* (%)	*N* (%)	*N* (%)
Comfort	Titanium	11 (73.3)	4 (26.7)	0	0	0
	Zirconia	6 (66.6)	3 (33.3)	0	0	0
Appearance	Titanium	13 (86.7)	2 (13.3)	0	0	0
	Zirconia	7 (77.8)	2 (22.2)	0	0	0
Chewing function	Titanium	14 (93.3)	1 (6.7)	0	0	0
	Zirconia	8 (88.9)	1 (11.1)	0	0	0
Taste perception	Titanium	13 (86.7)	2 (13.3)	0	0	0
	Zirconia	8 (88.9)	1 (11.1)	0	0	0
Fit	Titanium	12 (80)	3 (20)	0	0	0
	Zirconia	6 (66.7)	2 (22.2)	0	1 (11.1)	0
Overall satisfaction	Titanium	12 (80)	3 (20)	0	0	0
	Zirconia	7 (77.8)	2 (22.2)	0	0	0

Participation rates were higher among younger individuals. In the titanium group, 83.3% were very satisfied (overall satisfaction) and 16.7% were satisfied. In the zirconia group, 77.8% were very satisfied (overall satisfaction), 20.4% were satisfied, and 1.9% were dissatisfied. Due to the low response rate, no further statistical evaluation was performed.

## 4 Discussion

This study is the first RCT to directly compare zirconia‐ and titanium‐implant‐based all‐ceramic screw‐retained single implant supported crowns (SICs) up to 3 years in a valid clinical setting. It provides transparent and robust clinical data for SICs on two‐piece implant systems of comparable dimensions.

The primary objective of the non‐inferiority comparison in this study is to prove that the cumulative survival of titanium does not exceed that of zirconia by more than 10%. This was the basis for the sample size calculation. For this purpose, the one‐sided 95% CI was calculated for the empirical difference between the two cumulative survivals (titanium minus zirconia). To prove non‐inferiority, the upper limit of the CI would have to be less than 10%.

However, since the empirically estimated difference is already greater than 10%, the upper limit of the one‐sided 95% CI cannot be less than 10%. This means that there is no confidence that hybrid abutment SICs on zirconia implants will perform as well as SICs on titanium abutments, and care should be exercised when considering this treatment option for patient care.

If the alternative Hypothesis H1 could not be proven, this does not mean that the null hypothesis was proven. The Log‐Rank test for equality of both survival curves was not significantly rejected (*p* = 0.068).

Crowns were fabricated based on intraoperative scans in group titanium or splint registration in group zirconia obtained during implant placement. After implant healing, the final SIC was placed immediately after the second‐stage surgery (one abutment, one time procedure). Lithium disilicate crowns were chosen for their high flexural strength, aesthetic appearance, and reliable adhesive bonding on abutment luting‐bases (Naumann et al. 2023; Edelhoff et al. 2019).

Initial data on occlusal screw‐retained hybrid abutment crowns made of lithium disilicate on titanium implants are promising and show similar survival rates and rates of technical complications to our data (Mohseni et al. 2023; Naumann et al. 2023).

Three zirconia implants failed because of incomplete osseointegration early on due to a connective tissue cuff without bone contact with the implant but completely healed after revision and could be successfully restored. Implant loss within 24 months, attributed to problems with osseointegration of Zr implants, has been previously reported in various studies, as highlighted by Jank and Hochgatterer (2016), Koller et al. (2020), Brunello et al. (2022), Hossain et al. (2023), and Padhye et al. (2023). No further failure mode, such as implant fracture, was observed in this study, which aligns with the results of Zhang et al. (2022). While the healing time of 3 months for titanium implants can be regarded as the clinical standard, the evidence for ceramic implants is very limited. The manufacturer gives a recommendation of 3–6 months. The 4 months practiced in our study is therefore within the time frame recommended by the manufacturer and is comparable to other clinical studies (Kohal et al. 2020; Koller et al. 2020; Balmer et al. 2020).

Furthermore, two patients in the zirconia group were lost to follow‐up despite extensive efforts to locate them, adversely affecting the cumulative survival results. With a study sample of only 60 patients, each drop‐out or loss to follow‐up makes it more challenging to reach a scientifically reliable conclusion.

USPHS criteria serve as a strong parameter in assessing implant system functionality. We modified those used by Spies et al. (2019), who reported data on cemented crowns on one‐piece zirconia implants. Our results show similar functionality for both groups with no significant difference between or within the groups after 3 years observed. Since survival mainly depends on the implant, the modified USPHS criteria reflect the technical complications of the entire SIC complex, including the abutment itself.

While one screw loosening occurred in the titanium group and two in the zirconia group, an additional two ceramic crowns showed loss of retention from the PEKK base abutment. All SICs were placed at second‐stage surgery, the so‐called one abutment‐one time concept. This procedure may have caused the screw loosening, as tissue debris may have been trapped between the abutment and the implant shoulder during screwing, and the torque was reduced after resorption. The loss of retention between the lithium disilicate crown and the PEKK base abutment in the zirconia group shows that the micromechanical retention is a weak point for this specific abutment even though particle air‐abrasion used in this study exhibited the highest bond strength values (Cantarella et al. 2021). Successful repair was possible in all five cases. Loss of retention from the PEKK base underlines the need for a macro‐retentive design as the adhesive bond to PEKK remains a major challenge (Chopra et al. 2023; Arvai et al. 2024).

The PES, as defined by Fürhauser et al. (2005), was used for aesthetic evaluation in this study. Although initially intended for use in the anterior region, the diversity of the index allowed for a comprehensive assessment in the posterior region, particularly in connection with ceramic implants.

The PES parameters improved for both implant systems within 12 months after restoration. Although more clinically unacceptable values were initially observed in the zirconia group, the outcomes after 12 months were comparable. The results of this study suggest that the use of zirconia implants compared to titanium implants does not offer any significant benefits in terms of the visual appeal of the surrounding soft tissues when compared to titanium implants. Although assessing the results of PROMs without baseline data and with limited responses after 3 years poses a significant challenge, even if the majority of patients who responded were satisfied, due to the low response rate, no definite conclusion can be drawn. With the increasing importance of patient feedback, PROMs are now routinely administered, not only as part of this study but also as a standard practice of our dental clinic. This development reflects the growing recognition of the value of incorporating patient perspectives into evaluating the efficacy and quality of treatment.

Despite its strengths, this study had some limitations that warrant further attention. The number of 60 patients was relatively small, which may have limited the applicability of our findings to larger populations. Unequal dropout rates between the zirconia and titanium groups raise concerns about potential bias in the comparative outcomes. The results are strictly limited to the materials and components used and cannot therefore be generalised, particularly for the zirconia group. The evaluation of the aesthetic outcomes of the soft tissue assessed through PES/WES was conducted only after 1 year, limiting insights into the longitudinal aesthetic performance of the SICs. The absence of patient‐reported outcome measures at baseline and the limited response rate at 3 years may limit the comprehensiveness of the assessment.

The difference in the cumulative survival of the hybrid abutment SICs is not attributable to the prosthetic component, as no technical complications resulted in crown failure. In all three cases, aseptic, connective tissue septation of the implants was observed, which indicates incomplete osseointegration.

The primary objective of this study was to compare the cumulative survival and technical complications of screw‐retained hybrid abutment ceramic crowns on different implants, rather than to compare the implants themselves. It is, therefore, of the utmost importance to interpret the results in terms of their clinical significance. Although the *p*‐value showed no significant difference in cumulative survival rates, indicating no statistical dependence on the implant system, a clinically relevant difference is evident, with implant loss occurring in the zirconia group but not in the titanium group. This highlights that *p*‐value analysis can sometimes miss clinically important differences, such as the inferior survival rate of zirconia implants observed in the non‐inferiority analysis. On the one hand, the prosthetic component can be described as comparably successful for both groups. At the same time, a clinically significant difference in the rate of osseointegration can be observed at the implant level, with a total of six cases (three before crown SIC and three after baseline).

In conclusion, hybrid abutment SICs with PEKK base abutments on two‐piece zirconia implants could be an alternative to hybrid abutment SICs with titanium base on titanium implants. However, the lower osseointegration rate of the zirconia implants has to be considered.

## Author Contributions

**Guido Sterzenbach:** conceptualization, investigation, funding acquisition, writing – original draft, methodology, data curation, formal analysis. **Kristin Richter:** investigation, data curation, writing – original draft, formal analysis. **Klara Alpen:** investigation, data curation. **Hediyeh Khoshreza:** investigation, data curation. **Florian Beuer:** conceptualization, supervision. **Theodor Thiele:** conceptualization, investigation, methodology, funding acquisition, writing – review and editing, data curation.

## Ethics Statement

The study protocol was approved by the Ethical Committee of Charité‐Universitätsmedizin Berlin (number: EA4/041/18). The study was conducted in accordance with the Declaration of Helsinki and the Good Clinical Practice guidelines.

## Consent

Written informed consent was obtained from the study participants on a form approved by the Ethical Committee of Charité‐Universitätsmedizin Berlin, Germany.

## Conflicts of Interest

The authors declare no conflicts of interest.

## Data Availability

The data that support the findings of this study are available from the corresponding author upon reasonable request.
